# Photometric Stereo-Based Depth Map Reconstruction for Monocular Capsule Endoscopy

**DOI:** 10.3390/s20185403

**Published:** 2020-09-21

**Authors:** Yang Hao, Jing Li, Fei Meng, Peisen Zhang, Gastone Ciuti, Paolo Dario, Qiang Huang

**Affiliations:** 1 Intelligent Robotics Institute, School of Mechatronical Engineering, Beijing Institute of Technology, Beijing 100081, China; 3120150088@bit.edu.cn (Y.H.); 3120160106@bit.edu.cn (P.Z.); 2 Beijing Advanced Innovation Center for Intelligent Robots and Systems, Beijing Institute of Technology, Beijing 100081, China; 10902016@bit.edu.cn (J.L.); gastone.ciuti@santannapisa.it (G.C.); paolo.dario@santannapisa.it (P.D.); qhuang@bit.edu.cn (Q.H.); 3 Key Laboratory of Biomimetic Robots and Systems, Beijing Institute of Technology, Ministry of Education, Beijing 100081, China; 4 The BioRobotics Institute, Scuola Superiore Sant’Anna, 56025 Pisa, Italy; 5 Department of Excellence in Robotics & AI, Scuola Superiore Sant’Anna, 56025 Pisa, Italy

**Keywords:** monocular capsule endoscopy, photometric stereo, depth map reconstruction, non-ideal Lambertian surface, specular highlight

## Abstract

The capsule endoscopy robot can only use monocular vision due to the dimensional limit. To improve the depth perception of the monocular capsule endoscopy robot, this paper proposes a photometric stereo-based depth map reconstruction method. First, based on the characteristics of the capsule endoscopy robot system, a photometric stereo framework is established. Then, by combining the specular property and Lambertian property of the object surface, the depth of the specular highlight point is estimated, and the depth map of the whole object surface is reconstructed by a forward upwind scheme. To evaluate the precision of the depth estimation of the specular highlight region and the depth map reconstruction of the object surface, simulations and experiments are implemented with synthetic images and pig colon tissue, respectively. The results of the simulations and experiments show that the proposed method provides good precision for depth map reconstruction in monocular capsule endoscopy.

## 1. Introduction

The capsule endoscopy robot is a novel endoscopic device that can implement non-invasive digestive tract inspection. Limited by the dimension of the digestive tract, capsule endoscopy robots can only use monocular vision, which lacks depth perception. Therefore, depth map reconstruction is an important research topic for monocular capsule endoscopy robots [1,2,3,4].

Photometric stereo is a promising technology for depth map reconstruction in capsule endoscopy, since it only needs monocular vision and several light sources. For a Lambertian object surface, the relationship between surface depth and illuminance can be described by the photometric irradiance equation set based on the Lambertian reflection property [5], and the depth map can be reconstructed when the scale factor is known [6].

**Figure 1:**
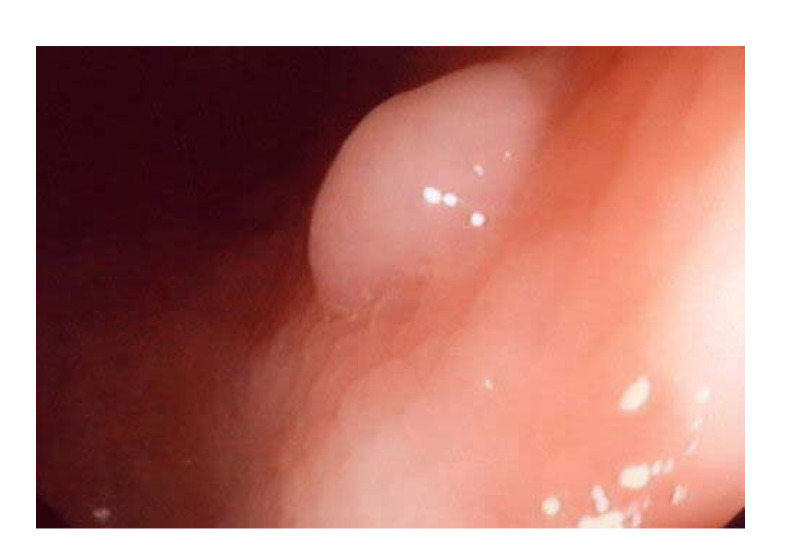
Digestive tract surface showing the specular highlight when illuminated by a light source from a certain direction.

However, to apply photometric stereo in monocular capsule endoscopy, there are two key problems to be solved. First, the object surface for endoscopy, i.e., the digestive tract, is not an ideal Lambertian surface. As shown in Figure 1, specular highlights always exist in the captured images; thus, photometric stereo cannot be applied directly for such object surfaces. Second, to reconstruct the depth map of the whole object surface with photometric stereo, the ground truth of the depth of at least one object surface point is needed to serve as a boundary condition.

To solve these problems, in this paper, we study the reflection property of the specular highlight on a non-ideal Lambertian surface and propose a method that estimates the depth of the specular highlight point and reconstructs the depth map of the digestive tract surface by photometric stereo.

The main contributions of this paper are: (1) we combine the specular property and Lambertian property of the object surface to estimate the depth of the specular highlight point, and (2) the estimated depth of the specular highlight point can serve as the boundary condition of the subsequent depth map reconstruction; therefore, no extra depth measurement devices are needed for the capsule endoscopy robot.

In Section 2, we introduce the state-of-the-art depth map reconstruction solutions for monocular capsule endoscopy. In Section 3, we propose a near point light source photometric stereo framework for the capsule endoscopy robot system. In Section 4, we present a depth map reconstruction method, which is based on the specular reflection constraint and photometric stereo. In Section 5 and Section 6, the proposed method is evaluated by simulation and experiment, respectively. The conclusion of this paper is given in Section 7.

## 2. Related Work

### 2.1. Depth Map Reconstruction in Capsule Endoscopy

Traditional methods for vision-based depth map reconstruction employed in minimally invasive surgery require the use of stereo cameras [7]. While present on modern stereo laparoscopes and robotic platforms, they are not found in capsule endoscopy systems due to physical constraints. Monocular 3D reconstruction techniques such as MonoSLAM [8] rely on completely static target structures, visual features that can be efficiently tracked, and a fast frame rate generating an abundance of viewpoints that can be used for structure recovery; a set of requirements that is not suitable for a typical capsule endoscopy setting. As a novel computer vision technology, machine learning has also been employed to explore the endoscopic and surgical applications [9,10,11]. However, training samples of endoscopy images need to be labeled with professional knowledge, and the measurement of the ground truth is also time consuming, which makes this route difficult to achieve currently.

Another vision-based 3D reconstruction method that has seen early applications to endoscopy and minimally invasive surgery is Shape-From-Shading (SFS) [12], where the 3D structure of the visualized scene is reconstructed from a single monocular camera without any further information required. Nevertheless, the reconstructed structures are in a metric space, i.e., they are linearly scaled with an unknown factor related to the surface albedo. Furthermore, the SFS problem is not a well-posed mathematical problem, which means it needs more restrictions to determine a unique solution for 3D reconstruction.

Photometric stereo is a monocular vision technology developed from SFS. By illuminating the object with multiple light sources from different directions, multiple irradiance equations can be obtained, and the well-posedness of the problem can be improved [13]. To employ photometric stereo in a capsule endoscopy application, an important property of the object surface needs to be noticed: the digestive tract is not an ideal Lambertian surface; covered by digestive juice, it shows specular reflection when the incident angle of illumination is close to the reflected angle. Therefore, to avoid the influence of the specular highlight in photometric stereo, researchers have explored different strategies.

### 2.2. Photometric Stereo-Based Depth Map Reconstruction of a Non-Ideal Lambertian Surface

The most straightforward strategy to avoid the specular highlight is to eliminate the specular highlight by adding external optical filters. From a physical point of view, visible light is a kind of electromagnetic wave with the polarization property. According to the Fresnel reflectance model, when the light is reflected, the specular component shows obvious directionality [14]. Based on this theory, Parot et al. added a pair of polarizers assembled in front of the light source and camera to reduce the specular reflection highlight [15]. However, the external polarization device is not good for minimizing the size ofcapsule endoscopy robots.

Another frequently-used strategy for the specular highlight is by separating the specular component of reflection by image processing. References [16,17] tried to eliminate the specular component in the pre-processing step. Dichromatic reflectance models have also been considered for diffuse and specular reflection separation. Reference [18] employed a dark channel prior, while [19] iteratively compared the intensity logarithmic differentiation of the input image. Yang and Abuja [20] used both the diffuse and specular components for the reconstruction based on the assumption that the illumination chromaticity is known and at least one of the input images is free of specularity. Nevertheless, these image processing-based methods are not robust enough when the amount of input images is not sufficient.

Except for eliminating or separating the specular component of reflection, some researchers also attempted to integrate the Lambertian and specular component into one general model. Ikehata et al. [21] used the purely diffuse irradiance equation for general surfaces, considered the specular component as a sparse error, and introduced a regression procedure, which requires tens of images to improve accuracy. However, the expensive algorithms based on energy minimization tend to be slow and cannot provide real-time shape reconstruction, and the light attenuation during propagation has not been considered. The Blinn–Phong model is an empirical light reflectance model that integrates diffuse reflection and specular reflection [22], and it is also studied for the photometric stereo problem of non-ideal Lambertian surfaces. Reference [23] combined the Blinn–Phong reflectance model and perspective projection to solve the 3D reconstruction problem; nevertheless, the infinite point light source assumption limits its usage for more general applications. Reference [24] integrated the Blinn–Phong reflectance model with the close point light source model; by performing iteration from one ground truth value of the surface depth, the surface depth map can be reconstructed. However, to reconstruct the whole object surface, extra devices are needed to measure the ground truth depth of the object surface as a boundary condition.

In this paper, we present a photometric stereo-based depth map reconstruction solution for a monocular capsule endoscopy robot, which makes use of the geometric property of specular highlight. The specular property and Lambertian property of the object surface are employed to estimate the depth of the specular highlight point, which can serve as the boundary condition of the subsequent depth map reconstruction.

## 3. Photometric Stereo Framework for the Capsule Endoscopy Robot

To ensure the precision of depth map reconstruction, an appropriate photometric stereo framework needs to be established based on the characteristics of the capsule endoscopy robot system. In this section, we first outline the basic structure of the capsule endoscopy robot system. Then, we propose a near point light source photometric stereo framework based on the characteristics of the capsule endoscopy robot.

### 3.1. Capsule Endoscopy Robot System

**Figure 2:**
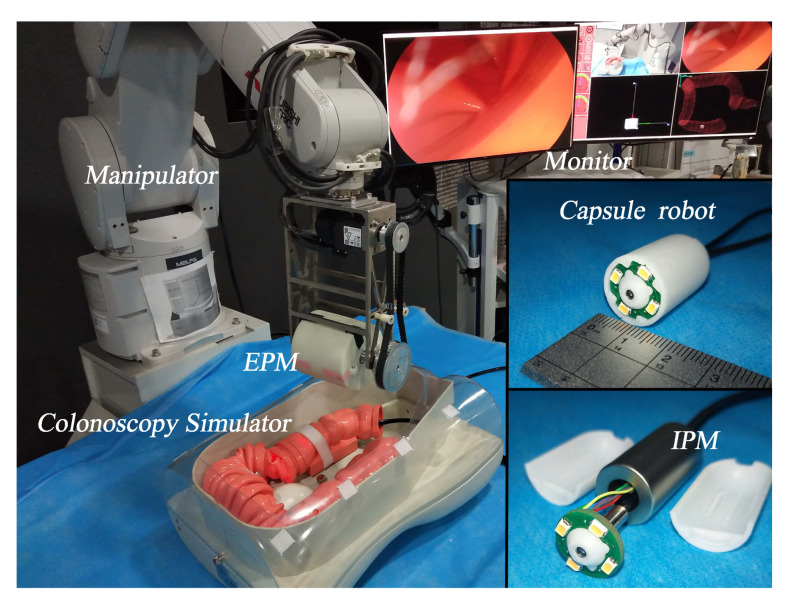
Magnetically-driven capsule endoscopy robot system. IPM, Internal Permanent Magnet; EPM, External Permanent Magnet.

Magnetic force can transmit without a medium, and it therefore has become the most frequently used driving force for capsule endoscopy robots [25,26,27]. A typical magnetically-driven capsule endoscopy robot system developed by the authors is shown in Figure 2. An Internal Permanent Magnet (IPM) is integrated in the capsule endoscopy robot as the locomotion unit, and an External Permanent Magnet (EPM) is attached at the end of a manipulator as a locomotion driver. By operating the EPM with the manipulator, the pose of the capsule endoscopy robot can be modified by magnetic force and torque.

The capsule endoscopy robot is designed to have an outer diameter of 14 mm and a length of 30 mm to ensure its maneuverability in the digestive tract of human beings [28]. The vision unit of the capsule robot is comprised of a monocular endoscopic camera, and it is located at the center of the capsule robot’s front side. The endoscopic camera has an ultra-short focal length, 640 × 480 pixels of resolution, and a 70.6 degree field of view and works with a depth of field from 3 mm to 100 mm. The illumination unit of the capsule endoscopy robot is comprised of four Prolight PS2P-TFPE FMR9 (3 mm × 2 mm × 0.8 mm, 0.2 W powered) surface-mounted LEDs, which are assembled around the camera with a 5.5 mm centrifugal distance. The four LEDs are driven by a TMS320F28335 DSP controller, and the illuminance of each light source can be modified independently by changing the duty cycle of the Pulse Width Modulation (PWM) signal.

### 3.2. Photometric Stereo Framework

For the capsule endoscopy robot system described in the previous part, photometric stereo is a promising solution for the depth map reconstruction of the object surface. Therefore, we propose a photometric stereo framework based on an endoscopic camera and surface-mounted LED light sources.

#### 3.2.1. Near Point Light Source Model

**Figure 3:**
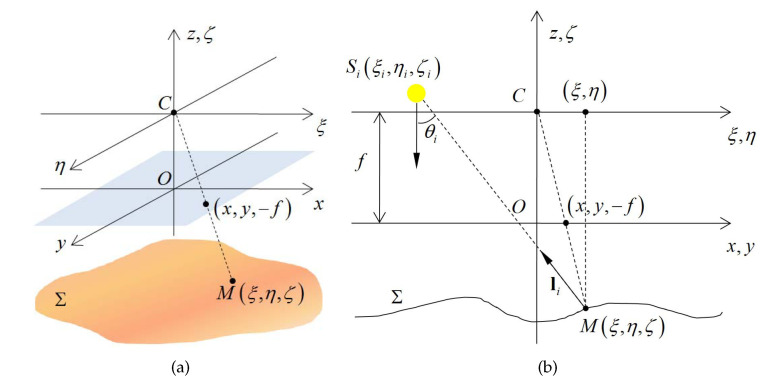
Photometric stereo framework. (**a**) Pinhole camera model of the endoscopic camera. (**b**) Near point light source model of the surface-mounted LED.

In order to provide the necessary ingredients of the photometric stereo framework, we first employ a pinhole camera model to describe the monocular endoscopic camera [5]. As shown in Figure 3a, the optical center of the camera is located at point *C*, and the Pixel Reference Frame (PRF) Oxyz is defined at the plane ξ=−f. For a given point *M* on the object surface Σ, it can be described by an unknown function $z\left(x,y\right)$ as:

(1) $$M\left(x,y\right)=\left(\xi ,\eta ,\zeta \right)=\left(-x\frac{z\left(x,y\right)}{f},-y\frac{z\left(x,y\right)}{f},z\left(x,y\right)\right),$$

where *f* is the focal length of the camera and the triple $\left(\xi ,\eta ,\zeta \right)=\left(\xi \left(x,y\right),\eta \left(x,y\right),\zeta \left(x,y\right)\right)$ comprises the coordinate in the Camera Reference Frame (CRF) Cξηζ.

Since the surface-mounted LEDs are close to the object surface, for different points on the object surface, the incident directions of illumination cannot be considered as parallel. Therefore, we employ the near point light source model to describe the surface-mounted LED light sources of the capsule endoscopy robot [12,29]. As shown in Figure 3b, when point *M* is illuminated by the surface-mounted LED source $S_{i}$ with position $\left(\xi_{i},\eta_{i},\zeta_{i}\right)$ (where $i\in \left[1,4\right]$), the direction of incident light can be given by vector $l_{i}\left(x,y,z\right)$, and the image of point *M* is projected at $\left(x,y,-f\right)$ on the Image Reference Frame (IRF).

The illumination direction vector $l_{i}\left(x,y,z\right)$ can be given by the ratio of direction vector ${\bar{l}}_{i}\left(x,y,z\right)$ and its module $q_{i}\left(x,y,z\right)$ as:

(2) $$l_{i}\left(x,y,z\right)=\frac{\left[\frac{\xi_{i}f}{z}+x,\frac{\eta_{i}f}{z}+y,\frac{\zeta_{i}f}{z}-f\right]}{\sqrt{{\left(\frac{\xi_{i}f}{z}+x\right)}^{2}+{\left(\frac{\eta_{i}f}{z}+y\right)}^{2}+{\left(\frac{\zeta_{i}f}{z}-f\right)}^{2}}}=\frac{{\bar{l}}_{i}\left(x,y,z\right)}{q_{i}\left(x,y,z\right)},$$

where:

(3) $${\bar{l}}_{i}\left(x,y,z\right)=\left[\frac{\xi_{i}f}{z}+x,\frac{\eta_{i}f}{z}+y,\frac{\zeta_{i}f}{z}-f\right],$$

(4) $$q_{i}\left(x,y,z\right)=\sqrt{{\left(\frac{\xi_{i}f}{z}+x\right)}^{2}+{\left(\frac{\eta_{i}f}{z}+y\right)}^{2}+{\left(\frac{\zeta_{i}f}{z}-f\right)}^{2}}.$$

#### 3.2.2. Light Source Attenuation

The illuminance of the surface-mounted LED light source is inhomogeneous and anisotropic; therefore, the attenuation of illuminance needs to be considered in the photometric stereo framework. The two main sources of illuminance attenuation are the propagation distance and the radial angle with respect to the principle direction [5].

**Figure 4:**
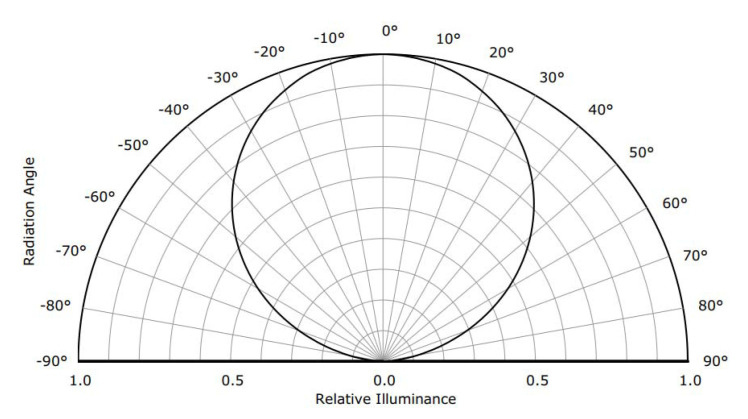
Radial attenuation of the surface-mounted LED with respect to the principle direction.

- Attenuation due to propagation distance:
  For a point light source, the energy absorbed by a given area on the object surface attenuates with respect to the inverse square of propagation distance. For light source $S_{i}$ and object point *M*, the illuminance attenuation factor $a_{i}^{\left(d\right)}\left(x,y,z\right)$ due to propagation distance can be described as:
  (5) $$a_{i}^{\left(d\right)}\left(x,y,z\right)=\frac{1}{{\left|{\mathrm{MS}}_{i}\right|}^{2}}=\frac{1}{{\left(\xi_{i}+x\frac{z}{f}\right)}^{2}+{\left(\eta_{i}+y\frac{z}{f}\right)}^{2}+{\left(\zeta_{i}-z\right)}^{2}}=\frac{f^{2}}{z^{2}q_{i}^{2}\left(x,y,z\right)}.$$
- Radial attenuation:
  The surface-mounted LED is a directional light source and has its maximal illuminance along a certain direction, which is known as the principle direction. When deviating from the principle direction, the illuminance of the surface-mounted LED usually attenuates with a cosine tendency with respect to the principle direction, as shown in Figure 4.
  Therefore, for light source $S_{i}$ and object point *M*, if we assume that the principle direction is along the −z direction, the radial attenuation factor $a_{i}^{\left(r\right)}\left(x,y,z\right)$ can be described as:
  (6) $$a_{i}^{\left(r\right)}\left(x,y,z\right)=cos\theta_{i}\left(x,y,z\right)=l_{i}\left(x,y,z\right)\cdot \left(0,0,1\right)=\frac{f}{q_{i}\left(x,y,z\right)}.$$

By combining the aforementioned two kinds of attenuations into a general illuminance attenuation factor, we get:

(7) $$a_{i}\left(x,y,z\right)=a_{i}^{\left(d\right)}\left(x,y,z\right)\cdot a_{i}^{\left(r\right)}\left(x,y,z\right)=\frac{f^{3}}{z^{2}q_{i}^{3}\left(x,y,z\right)}$$

## 4. Photometric Stereo-Based Depth Map Reconstruction

In this section, we introduce the depth map reconstruction method for the capsule endoscopy robot based on the photometric stereo framework proposed in the previous section. First, we analyze the property of the object surface of the capsule endoscope and introduce the reflection model of the non-ideal Lambertian surface. Then, we propose a method that combines the specular property and Lambertian property of reflection to estimate the depth of the specular highlight point. Finally, we introduce the depth map reconstruction method based on the depth estimation of the specular highlight point.

### 4.1. Reflection Model of the Non-Ideal Lambertian Surface

The object surface of the capsule endoscope is generally the inner wall of the digestive tract of human beings, and it is always covered by a film of digestive juice; thus, it has a two-fold reflection property: it can show both the specular property and Lambertian property, depending on whether the incident angle is close to the reflected angle or not.

**Figure 5:**
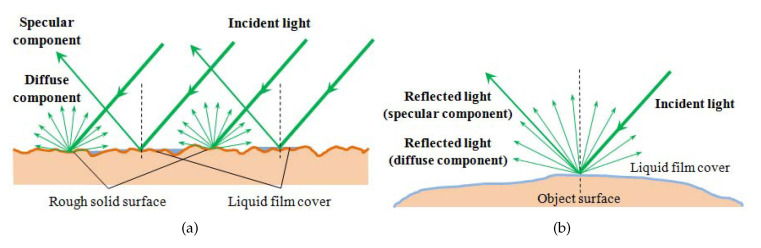
Lambertian reflection and specular reflection on the digestive tract. (**a**) Reflection on the digestive tract surface observed from a microscopic viewpoint. (**b**) General reflection model of the non-ideal Lambertian surface.

As shown in Figure 5a, from a microscopic viewpoint, the surface of the digestive tract is not flat. On the one hand, the concave parts of the surface can be covered by liquid more easily and thus show significant specular reflection when illuminated by incident light. The convex parts, on the other hand, show more Lambertian reflection because of the roughness.

Therefore, from a macroscopic viewpoint, because the concave parts and convex parts of the liquid covered surface are distributed evenly, we can unify the specular reflectance and Lambertian reflectance on the object surface into a general reflection model, as shown in Figure 5b, in which the concave parts contribute the specular reflection component along a certain direction, while the convex parts contribute the Lambertian reflection component along all outward directions. Note that, though our model is similar to the Blinn–Phong reflection model, the difference is that our model discusses the geometric property of the specular component, i.e., the incident angle equals the reflected angle, which is different from the photometric property of the specular component discussed in the Blinn–Phong model.

**Figure 6:**
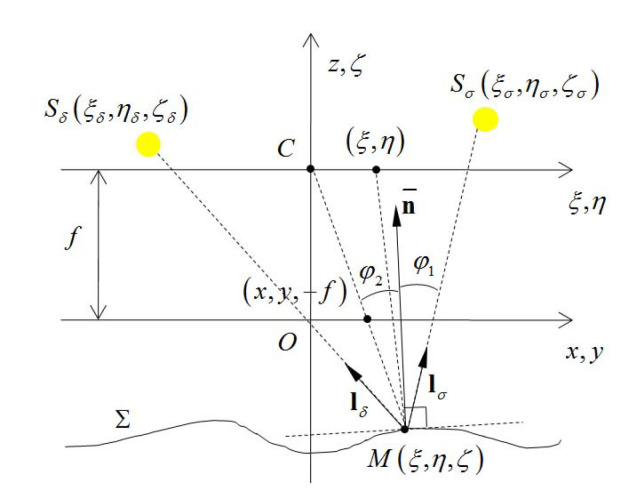
Lambertian reflection and specular reflection under illumination from different light sources. The light source $S_{\delta }$ creates Lambertian reflection, while the light source $S_{\sigma }$ creates specular reflection.

To parameterize the aforementioned reflection model, we define a scenario in which the specular reflection and Lambertian reflection are created by different light sources, respectively, as shown in Figure 6.

For point *M* on the object surface Σ, the normalized outgoing normal vector $n\left(x,y\right)$ can be given by:

(8) $$n\left(x,y\right)=\frac{\bar{n}\left(x,y\right)}{\left|\bar{n}\left(x,y\right)\right|},$$

where:

(9) $$\bar{n}\left(x,y\right)=\frac{z}{f^{2}}\left[f\nabla z\left(x,y\right),z\left(x,y\right)+\left(x,y\right)\cdot \nabla z\left(x,y\right)\right].$$

For the ease of notation, here, we assume that among the four point light sources of the photometric stereo framework, the light source $S_{\sigma }$ creates the specular highlight at point *M*, while the other three light sources $S_{\delta_{1}}$ to $S_{\delta_{3}}$ create Lambertian reflection at point *M*. Then, we derive the two key equations in this paper, i.e., the specular reflection equation and the photometric irradiance equation set, from the specular reflection constraint and Lambertian reflection property.

- Specular reflection equation:
  For light source $S_{\sigma }$, because the incident angle equals the reflected angle, we get the specular reflection equation as:
  (10) $$\frac{\mathrm{MC}}{\left|\mathrm{MC}\right|}+\frac{{\bar{l}}_{\sigma }\left(x,y,z\right)}{q_{\sigma }\left(x,y,z\right)}=\lambda \bar{n}\left(x,y\right),$$
  where:
  (11) $$\mathrm{MC}=\left[x\frac{z}{f},y\frac{z}{f},-z\right]$$
  and λ is a scale factor. Then, by substituting the terms $\bar{n}\left(x,y\right)$ and MC in the specular reflection equation with the expressions in Equations (9) and (11), the specular reflection equation can be further notated as:
  (12) $$\frac{\left[x,y,-f\right]}{\sqrt{x^{2}+y^{2}+f^{2}}}+\frac{{\bar{l}}_{\sigma }\left(x,y,z\right)}{q_{\sigma }\left(x,y,z\right)}=\lambda \frac{z}{f^{2}}\left[fz_{x},fz_{y},z+xz_{x}+yz_{y}\right].$$
- Photometric irradiance equation set:
  For light source $S_{\delta_{1}}$ to $S_{\delta_{3}}$, because of the Lambertian reflection, the photometric irradiance equation set can be obtained as:
  (13) $$\left\{\begin{array}{c} I_{\delta_{1}}\left(x,y\right)=a_{\delta_{1}}\left(x,y,z\right)\rho \left(x,y\right)\left(l_{\delta_{1}}\left(x,y,z\right)\cdot n\left(x,y\right)\right)\\ I_{\delta_{2}}\left(x,y\right)=a_{\delta_{2}}\left(x,y,z\right)\rho \left(x,y\right)\left(l_{\delta_{2}}\left(x,y,z\right)\cdot n\left(x,y\right)\right)\\ I_{\delta_{3}}\left(x,y\right)=a_{\delta_{3}}\left(x,y,z\right)\rho \left(x,y\right)\left(l_{\delta_{3}}\left(x,y,z\right)\cdot n\left(x,y\right)\right) \end{array}\right.$$
  where $a_{\delta }\left(x,y,z\right)$ is the attenuation factor and $\rho \left(x,y\right)$ is the albedo of the object surface. By substituting the terms $l_{\delta }\left(x,y,z\right)$, $n\left(x,y\right)$ and the attenuation factor $a_{\delta }\left(x,y,z\right)$ with the expressions in Equations (2), (7), and (8), the photometric irradiance equation set Equation (13) can be notated as:
  (14) $$\left\{\begin{array}{c} I_{\delta_{1}}\left(x,y\right)=\frac{f^{3}\rho \left(x,y\right)}{z^{2}\left|\bar{n}\left(x,y\right)\right|}\cdot \frac{\left({\bar{l}}_{\delta_{1}}\left(x,y,z\right)\cdot \bar{n}\left(x,y\right)\right)}{q_{\delta_{1}}^{4}\left(x,y,z\right)}\\ I_{\delta_{2}}\left(x,y\right)=\frac{f^{3}\rho \left(x,y\right)}{z^{2}\left|\bar{n}\left(x,y\right)\right|}\cdot \frac{\left({\bar{l}}_{\delta_{2}}\left(x,y,z\right)\cdot \bar{n}\left(x,y\right)\right)}{q_{\delta_{2}}^{4}\left(x,y,z\right)}\\ I_{\delta_{3}}\left(x,y\right)=\frac{f^{3}\rho \left(x,y\right)}{z^{2}\left|\bar{n}\left(x,y\right)\right|}\cdot \frac{\left({\bar{l}}_{\delta_{3}}\left(x,y,z\right)\cdot \bar{n}\left(x,y\right)\right)}{q_{\delta_{3}}^{4}\left(x,y,z\right)}. \end{array}\right.$$

### 4.2. Depth Estimation of Specular Highlight Point

To estimate the depth of specular highlight point *z*, we construct a Partial Derivative Equation (PDE) about *z* from the photometric irradiance equation set, Equation (13), and eliminate its partial derivative terms $z_{x}$ and $z_{y}$ by combining it with the specular reflection Equation (11).

First, for the photometric irradiance equation set, we eliminate the term $\frac{f^{3}\rho \left(x,y\right)}{z^{2}\left|\bar{n}\left(x,y\right)\right|}$ by making ratios between different photometric irradiance equations, and an image ratio equation can be obtained as:

(15) $$\frac{I_{\delta_{1}}\left(x,y\right)}{I_{\delta_{2}}\left(x,y\right)}=\frac{q_{\delta_{2}}^{4}\left(x,y,z\right)}{q_{\delta_{1}}^{4}\left(x,y,z\right)}\cdot \frac{\left({\bar{l}}_{\delta_{1}}\left(x,y,z\right)\cdot \bar{n}\left(x,y\right)\right)}{\left({\bar{l}}_{\delta_{2}}\left(x,y,z\right)\cdot \bar{n}\left(x,y\right)\right)}.$$

For the ease of notation, we denote the terms $q_{i}\left(x,y,z\right)$ and $I_{i}\left(x,y\right)$ as $q_{i}$ and $I_{i}$ in the following expressions, respectively. Considering that:

(16) $$\begin{array}{cc} {\bar{l}}_{\delta }\left(x,y,z\right)\cdot \bar{n}\left(x,y\right) & =\frac{z}{f^{2}}\cdot \left[\frac{\xi_{\delta }f}{z}+x,\frac{\eta_{\delta }f}{z}+y,\frac{\zeta_{\delta }f}{z}-f\right]\cdot \left[f\nabla z\left(x,y\right),z\left(x,y\right)+\left(x,y\right)\cdot \nabla z\left(x,y\right)\right]\\ & =\left(\xi_{\delta }+\frac{\zeta_{\delta }}{f}x\right)z_{x}+\left(\eta_{\delta }+\frac{\zeta_{\delta }}{f}y\right)z_{y}+\left(\zeta_{\delta }-z\right)\frac{z}{f}, \end{array}$$

a PDE about depth *z* can be notated as:

(17) $$F_{\xi ,\delta_{1,2}}\left(x,y,z\right)\cdot z_{x}+F_{\eta ,\delta_{1,2}}\left(x,y,z\right)\cdot z_{y}=z\cdot F_{\zeta ,\delta_{1,2}}\left(x,y,z\right),$$

where:

(18) $$\left\{\begin{array}{c} F_{\xi ,\delta_{1,2}}\left(x,y,z\right)=I_{\delta_{1}}q_{\delta_{1}}^{4}\left(\xi_{\delta_{2}}f+\zeta_{\delta_{2}}x\right)-I_{\delta_{2}}q_{\delta_{2}}^{4}\left(\xi_{\delta_{1}}f+\zeta_{\delta_{1}}x\right)\\ F_{\eta ,\delta_{1,2}}\left(x,y,z\right)=I_{\delta_{1}}q_{\delta_{1}}^{4}\left(\eta_{\delta_{2}}f+\zeta_{\delta_{2}}y\right)-I_{\delta_{2}}q_{\delta_{2}}^{4}\left(\eta_{\delta_{1}}f+\zeta_{\delta_{1}}y\right)\\ F_{\zeta ,\delta_{1,2}}\left(x,y,z\right)=I_{\delta_{2}}q_{\delta_{2}}^{4}\left(\zeta_{\delta_{1}}-z\right)-I_{\delta_{1}}q_{\delta_{1}}^{4}\left(\zeta_{\delta_{2}}-z\right). \end{array}\right.$$

Then, to eliminate the partial derivative terms in Equation (17), we derive the relationship between depth *z* and partial derivatives $z_{x}$ and $z_{y}$ from the specular reflection Equation (12). By substituting ${\bar{l}}_{\sigma }\left(x,y,z\right)$ with the expression in Equation (3), the scale factor λ can be eliminated, and after some algebra, two expressions about $z_{x}$ and $z_{y}$ can be obtained as:

(19) $$z_{x}=-\frac{xzq_{\sigma }\left(x,y,z\right)+\left(xz+\xi_{\sigma }f\right)b\left(x,y\right)}{b\left(x,y\right)\left(q_{\sigma }\left(x,y,z\right)+b^{2}\left(x,y\right)+f\frac{x\xi_{\sigma }+y\eta_{\sigma }-f\zeta_{\sigma }}{z}\right)}$$

and:

(20) $$z_{y}=-\frac{yzq_{\sigma }\left(x,y,z\right)+\left(yz+\eta_{\sigma }f\right)b\left(x,y\right)}{b\left(x,y\right)\left(q_{\sigma }\left(x,y,z\right)+b^{2}\left(x,y\right)+f\frac{x\xi_{\sigma }+y\eta_{\sigma }-f\zeta_{\sigma }}{z}\right)},$$

where:

(21) $$b\left(x,y\right)=\sqrt{x^{2}+y^{2}+f^{2}}.$$

Finally, by substituting the partial derivative terms $z_{x}$ and $z_{y}$ in Equation (17) with the expressions in Equations (19) and (20), we get:

(22) $$F_{\xi ,\delta_{1,2}}\left(x,y,z\right)\cdot G_{\xi ,\sigma }\left(x,y,z\right)+F_{\eta ,\delta_{1,2}}\left(x,y,z\right)\cdot G_{\eta ,\sigma }\left(x,y,z\right)=-F_{\zeta ,\delta_{1,2}}\left(x,y,z\right)\cdot G_{\zeta ,\sigma }\left(x,y,z\right)$$

where:

(23) $$\left\{\begin{array}{c} G_{\xi ,\sigma }\left(x,y,z\right)=xzq_{\sigma }+\left(xz+\xi_{\sigma }f\right)b\left(x,y\right)\\ G_{\eta ,\sigma }\left(x,y,z\right)=yzq_{\sigma }+\left(yz+\eta_{\sigma }f\right)b\left(x,y\right)\\ G_{\zeta ,\sigma }\left(x,y,z\right)=zq_{\sigma }b^{2}\left(x,y\right)+zb^{3}\left(x,y\right)+f\left(x\xi_{\sigma }+y\eta_{\sigma }-f\zeta_{\sigma }\right)b\left(x,y\right). \end{array}\right.$$

Since the only unknown of Equation (22) is the depth *z* of the specular highlight point, we move all terms to the same side of the equation and obtain an energy function as:

(24) $$\begin{array}{cc} \; & E\left(x,y,z\right)\\ & =∥F_{\xi ,\delta_{1,2}}\left(x,y,z\right)\cdot G_{\xi ,\sigma }\left(x,y,z\right)+F_{\eta ,\delta_{1,2}}\left(x,y,z\right)\cdot G_{\eta ,\sigma }\left(x,y,z\right)+F_{\zeta ,\delta_{1,2}}\left(x,y,z\right)\cdot G_{\zeta ,\sigma }\left(x,y,z\right)∥. \end{array}$$

By solving the minimization problem of this energy function, the depth *z* of the specular highlight point can be estimated.

### 4.3. Depth Map Reconstruction of the Object Surface

To reconstruct the depth map of the whole object surface, we consider solving the Partial Differential Equation (PDE) (17). Since the PDE (17) has three unknowns *z*, $z_{x}$, and $z_{y}$, the unique solution cannot be obtained with only one Dirichlet boundary condition. Therefore, to improve the well-posedness of this problem, we extend it to an equation set based on three image ratio equations, then an iteration scheme can be obtained [5]. By using the estimated depth of the specular highlight point as the boundary condition (seed point) of the iteration scheme, the depth map of the whole object surface can be then reconstructed.

In order to simplify the notation, we denote Equation (17) as:

(25) $$F_{\delta_{1},\delta_{2}}\left(x,y,z\right)\cdot \nabla z=s_{\delta_{1},\delta_{2}}\left(x,y,z\right),$$

where:

(26) $$F_{\delta_{1},\delta_{2}}\left(x,y,z\right)=\left[F_{\xi ,\delta_{1,2}}\left(x,y,z\right),F_{\eta ,\delta_{1,2}}\left(x,y,z\right)\right]$$

and:

(27) $$s_{\delta_{1},\delta_{2}}\left(x,y,z\right)=z\cdot F_{\zeta ,\delta_{1,2}}\left(x,y,z\right).$$

In the discretization of the PDEs, we define the position of point *M* to be described by discrete spatial coordinate $\left(i,j\right)$ as $\left(x_{i},y_{j},Z_{j,i}\right)$, and the step length along the *x* and *y* directions as $\Delta_{x}$ and $\Delta_{y}$, respectively, then the forward upwind scheme can be obtained as:

(28) $$\begin{array}{cc} \; & F_{\xi ,\delta_{1,2}}\left(i,j\right)\frac{Z_{i+1,j}-Z_{i-1,j}}{2\Delta_{x}}+F_{\eta ,\delta_{1,2}}\left(i,j\right)\frac{Z_{i,j+1}-Z_{i,j-1}}{2\Delta_{y}}\\ \; & =\left|F_{\xi ,\delta_{1,2}}\left(i,j\right)\right|\frac{\Delta_{x}}{2}\frac{Z_{i+1,j}-2Z_{i,j}+Z_{i-1,j}}{\Delta_{x}^{2}}+\left|F_{\eta ,\delta_{1,2}}\left(i,j\right)\right|\frac{\Delta_{y}}{2}\frac{Z_{i,j+1}-2Z_{i,j}+Z_{i,j-1}}{\Delta_{y}^{2}}+s\left(i,j\right). \end{array}$$

The artificial diffusion introduced in the right-hand side of Equation (28) allows us to follow the vector field F by considering the most appropriate discretization for the first derivative in order to track the characteristic lines [30,31]. In particular, it is a consistent numerical scheme of order equal to one with respect to both $\Delta_{x}$ and $\Delta_{y}$. After some algebra, Equation (28) can be simplified as:

(29) $$\begin{array}{cc} \; & \left|F_{\xi ,\delta_{1,2}}\left(i,j\right)\right|Z_{i-sgn\left(F_{\xi ,\delta_{1,2}}\left(i,j\right)\right),j}+\left|F_{\eta ,\delta_{1,2}}\left(i,j\right)\right|Z_{i,j-sgn\left(F_{\eta ,\delta_{1,2}}\left(i,j\right)\right)}\\ & =Z\left(i,j\right)\left(\left|F_{\xi ,\delta_{1,2}}\left(i,j\right)\right|+\left|F_{\eta ,\delta_{1,2}}\left(i,j\right)\right|\right)-\Delta s_{\delta_{1},\delta_{2}}\left(i,j\right), \end{array}$$

where $\Delta_{x}=\Delta_{y}=\Delta$, and we emphasize again the dependence of the functions F and *s* on *z*. Using a fast-marching procedure, we couple the pairs of Equation (29) from different light source pairs in order to compute the directional derivative according to eight principal directions (2 horizontals, 2 verticals, and 4 diagonals), which span the two-dimensional image domain [32]. To reduce the error, we chose the specific equation by taking the pair of images that have the highest gray scale value at pixel $\left(x_{i},y_{j}\right)$.

## 5. Simulation

The proposed depth estimation method for the specular highlight point in the previous section is based on the assumption that the incident angle equals the reflected angle. Nevertheless, in practical scenarios, the specular highlight is not always displayed as a certain point with the ideal specular property, but as a highlight region in which the pixels show the specular property in different degrees, i.e., the incident angle may not exactly equal the reflected angle. For this reason, the depth estimation errors of pixels in the specular highlight region are different from each other, and this can further influence the precision of the subsequent depth map reconstruction of the whole object surface. Therefore, to evaluate the effectiveness of the proposed method in a more practical scenario, we define a photometric stereo framework of monocular capsule endoscopy in a simulation environment and conduct simulation tests.

### 5.1. Simulation Configuration

**Figure 7:**
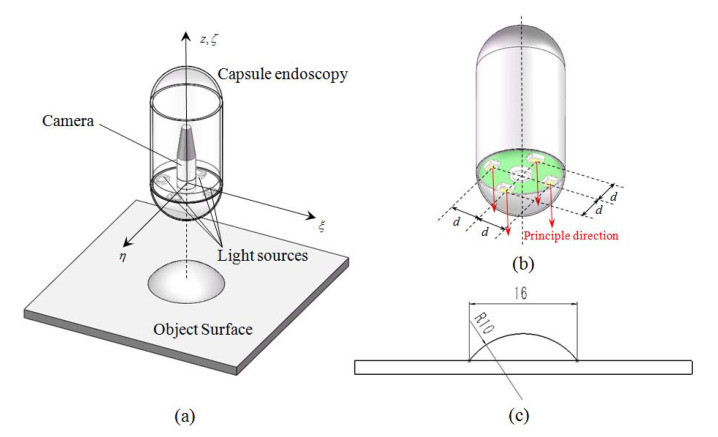
Simulation configuration of photometric stereo in monocular capsule endoscopy. (**a**) General view of the simulation environment. (**b**) Camera and light sources’ distribution in the photometric stereo frame (where *d* is the distance from the camera to each light source). (**c**) Section view of the object surface (dimensions in millimeters).

A general view of our simulation configuration is given in Figure 7a. Here, we introduce the details of configuration in three parts: photometric stereo framework, object surface, and optical environment.

#### 5.1.1. Photometric Stereo Framework

As shown in Figure 7b, the photometric stereo framework consists of a camera and four surface-mounted LEDs.

The camera is defined as an ideal pinhole camera, i.e., there is no geometric, chromatic, or photometric aberration during the imaging procedure. The focal length of the camera is defined as 565 pixels, the same as that in real experiments.

The four surface-mounted LED light sources are defined as near point light sources with two sources of illuminance attenuation, and they are placed surrounding the camera with the same distance *d* to the camera. To simplify the calculation, we assume that the four light sources and the camera are in the same plane, and the illuminance of the four light sources is the same. The principle directions of the light sources are all along the principle direction of the camera, and the distance *d* from the camera to each light source is defined as 5.5 mm. To obtain color images, we define the light sources in simulation to emit white light.

For the image processing configuration, we assume that all images created in the simulation environment are well exposed, and all image post-processing procedures, e.g., gamma correction, contrast, saturation, brightness, etc., are all closed; therefore, the pixel intensity in the generated images is proportional to the illuminance of the light sources.

#### 5.1.2. Object Surface

Digestive tract polyps are the most typical objects for capsule endoscopy screening. Therefore, in our simulation environment, we define a polyp-shaped object surface to evaluate the precision of the proposed method. The shape and dimension of the object surface are shown in Figure 7c. The surface with a convex profile prevents from shadow and self-blocking, and the diameter of the spherical part is defined with reference to the real digestive tract polyps of human beings.

To simulate the reflectance of a liquid-covered rough surface in a practical scenario, we use the Blinn–Phong model to describe the reflectance of the object surface [22]. The factor for glossiness in the Blinn–Phong model is elaborately selected; therefore, the distribution of the specular highlight region is close to that in real experiments. The color of the object surface is defined as a real human digestive tract.

Since the digestive tract of human beings has homogeneous reflectance, we define that the reflectance of the object surface is also homogeneous. To optimize the focus of the endoscopic camera, we place the object surface aligned with the photometric stereo framework, and we define the distance from the camera to the object surface as 21.37 mm in the simulation and the subsequent real experiments.

#### 5.1.3. Optical Environment

To simulate the dark environment in the digestive tract of human beings, we define the photometric stereo framework and the object surface to be isolated from external lighting. All illuminations in the simulation environment only come from the surface-mounted LEDs of the photometric stereo framework, and they can only be reflected by the object surface.

We further define that the secondary reflection does not occur on the object surface, and the propagation medium of light in the simulation is transparent and homogeneous.

### 5.2. Simulation Procedures and Results

#### 5.2.1. Image Generation and Specular Highlight Region Detection

**Figure 8:**
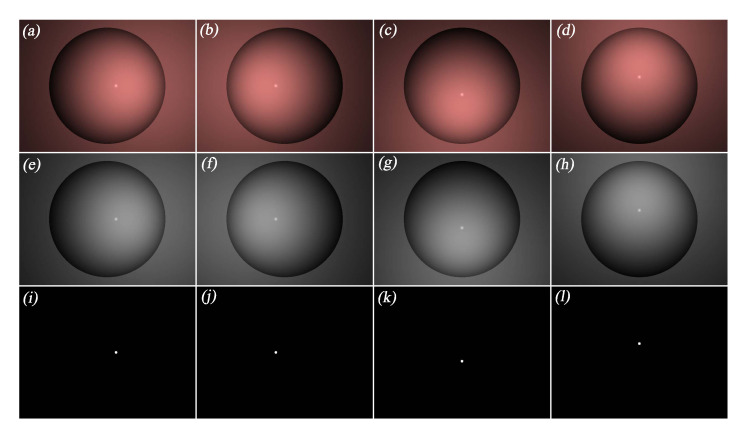
Photometric stereo images, illuminance maps, and detected specular highlight regions in the simulation environment. (**a**–**d**): generated photometric stereo images in the simulation environment. (**e**–**h**): illuminance maps of the photometric stereo images in the simulation environment. (**i**–**l**): detected specular highlight regions in the simulation environment.

Based on the aforementioned simulation configuration, four photometric stereo images were generated by successively enabling the four light sources, as shown in the first row of Figure 8. The illuminance maps of the generated photometric stereo images are then obtained, as shown in the second row of Figure 8.

Considering that the specular highlight regions have low saturation and high intensity [2], we set the thresholds of saturation and intensity for detecting specular highlight in the four images, and the results of the detection are shown in the third row of Figure 8.

#### 5.2.2. Depth Estimation of Specular Highlight Points

**Figure 9:**
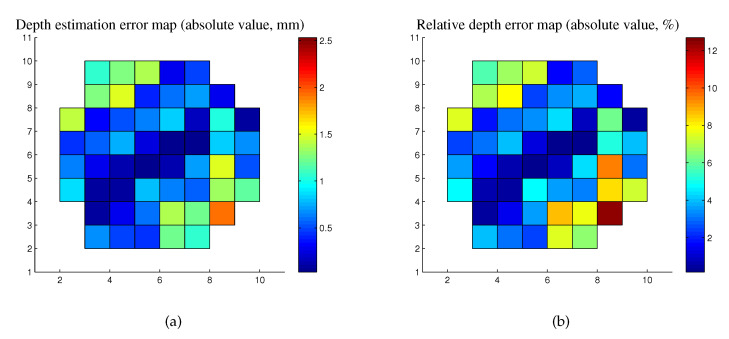
Depth estimation error distribution of the specular highlight region. (**a**) Depth estimation error map (absolute value). (**b**) Relative depth error map (absolute value).

For all pixels in the detected specular highlight regions, we conducted the proposed depth estimation method. Since the four generated images are symmetric to each other, here we only discuss the result of Figure 8a. The depth estimation error of each specular pixel is obtained by comparing the estimation value and the ground truth, and the depth estimation error distribution of the specular highlight region is shown in Figure 9. Note that the relative depth errors are obtained by calculating the ratios of the absolute values of depth estimation errors to the ground truth depth of each specular highlight pixel.

According to the obtained result, the pixel at the centroid of the specular highlight region has the minimal depth estimation error 0.0488 mm and relative depth error 0.2808%, which come from the estimated depth −17.3700 mm and ground truth −17.4188 mm. The Root Mean Squared Error (RMSE) of the depth estimation of all pixels in the specular highlight region is 0.9355 mm, and the corresponding relative depth error is 5.3857%. Obviously, the depth estimation of the centroid pixel of the specular highlight region is the most suitable boundary condition for the subsequent depth map reconstruction, since it has the best estimation of depth among all points in the specular highlight region.

#### 5.2.3. Depth Map Reconstruction of the Object Surface

**Figure 10:**
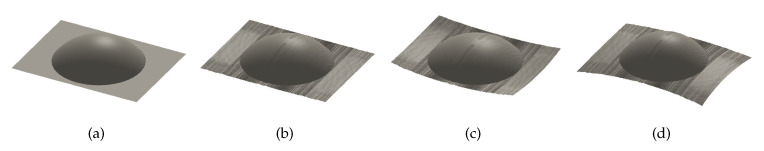
Results of the depth map reconstruction tests based on the specular highlight pixels with different depth estimation precisions in the simulation. (**a**) Ground truth of the object surface. (**b**) Depth map reconstruction results based on the specular highlight pixel with minimal depth estimation error. (**c**) Depth map reconstruction results based on the specular highlight pixel with positive RMS depth estimation error. (**d**) Depth map reconstruction results based on the specular highlight pixel with negative RMS depth estimation error.

To evaluate how the depth estimation error of the specular highlight point influences the precision of subsequent depth map reconstruction, we use the depth estimation results of the specular highlight pixels in Section 5.2.2 as the boundary conditions of the forward upwind scheme in Section 4.3. The depth map reconstruction tests were implemented based on specular highlight pixels with minimal depth estimation error and the RMSE of depth estimation. The results of the depth map reconstruction tests are shown in Figure 10 and Table 1.

For the centroid of the specular highlight region, i.e., the specular highlight pixel with minimal depth estimation error, the reconstructed depth map of the object surface almost has the same shape of the ground truth; the RMSE of the depth reconstruction of the object surface is less than 0.1 mm; and the relative RMSE of the depth reconstruction of the object surface is less than 0.5%, which is relatively precise for the capsule endoscopy application.

For the specular highlight pixels with the RMSE of depth estimation, the RMSEs of the reconstructed depth map of the object surface are less than 2 mm, and the relative RMSEs of the object surface depth reconstruction are less than 10%, which are also acceptable for the capsule endoscopy application.

## 6. Experiment

To further evaluate the effectiveness of the proposed method for the practical monocular capsule endoscopy robot, we conducted a depth map reconstruction experiment with pig colon tissue.

### 6.1. Experiment Devices

**Figure 11:**
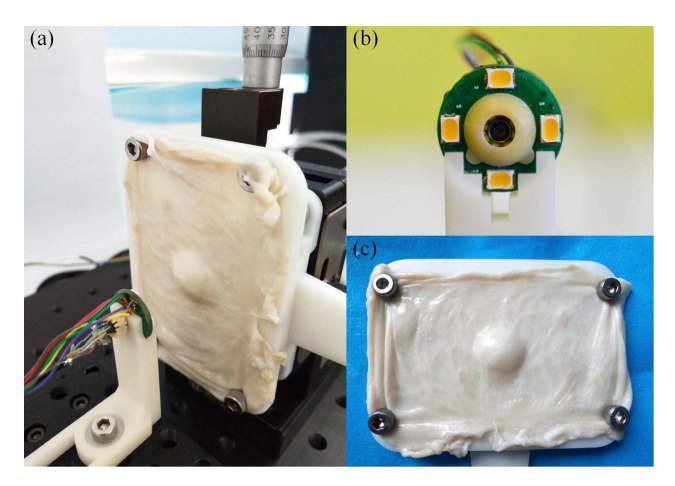
Test bench for the experiment. (**a**) General view of the test bench. (**b**) Front view of the monocular capsule endoscope. (**c**) Pig colon tissue fixed on the 3D printed base board. (Considering the institutional and governmental regulations about the ethical use of animals, all pig colon tissues used in this study are bought from market, as food grade.)

To guarantee the dimensional precision of the experiment, we established a test bench for depth map reconstruction, as shown in Figure 11a.

The monocular vision unit and illumination unit of the capsule endoscopy robot are fixed on a 2D linear stage with a 3D printed holder, as shown in Figure 11b. The pig colon tissue is fixed on a 3D printed base board to prevent from deformation, as shown in Figure 11c. For comparison with the simulation tests in Section 4, the profile of the 3D printed base board is designed with the same shape and dimensions as those in Figure 7c.

### 6.2. Experiment Configuration

#### 6.2.1. Photometric Stereo Framework Calibration

Different from the ideal models in the simulation, the camera and light sources in the practical photometric stereo framework need to be calibrated before experiments.

- Camera calibration:
  For the practical endoscopic camera, different kinds of aberrations exist in the imaging system, and they can be classified into two main categories: geometric aberration and photometric aberration.
  The geometric aberration of the camera is caused by the inherent optical property and assembling error of the spherical lens. To compensate the geometric aberration, the internal parameters of the camera must be calibrated first. By calibration with a chessboard pattern [33], the intrinsic parameters of the endoscopic camera are obtained as shown in Table 2. The average reprojection error of the calibration is 0.157224 pixels.
  The photometric aberration of the camera is caused by the post-processing procedures of the image sensor. Procedures such as gamma correction, modification of saturation, contrast, white balance, etc., can make the pixel intensity in the image not proportional to the incoming illuminance. To obtain the precise illuminance map of each image, we calibrate the non-linear relationship between the illuminance on the image sensor and the pixel intensity in the captured images.
- Light source calibration:
  The surface-mounted LED light sources need to be calibrated, as their illuminance and assembling are not perfect.
  With respect to illuminance, though all LED light sources in the photometric stereo framework are powered by the same voltage, the illuminance of each LED may still be different since the diode voltage drop of each individual LED is different. Therefore, it is necessary to calibrate the illuminance difference between the LED light sources.
  With respect to assembling, errors in the position and principle direction of the LED light sources are inevitable. Therefore, to obtain more precise positions and principle directions of the light sources, we employ the light source position calibration method and principle direction calibration method in [34,35] to calibrate the light sources.

#### 6.2.2. Optical and Imaging Preparation

- External light shield:
  To avoid the disturbance of external light, all experiments were conducted in a black box.
- Exposure
  To ensure that the captured images were well exposed, we closed the auto exposure function of the camera and matched the illuminance of the light sources with the camera’s exposure setting by modifying the duty cycle of the PWM signal.
- Image post-processing:
  Since the photometric-based method is very sensitive to the noise of illuminance, the Gaussian filter and medium filter are used in image post-processing to decrease the white noise and salt-and-pepper noise.

### 6.3. Experiment Procedure and Results

#### 6.3.1. Image Capture and Specular Highlight Region Detection

**Figure 12:**
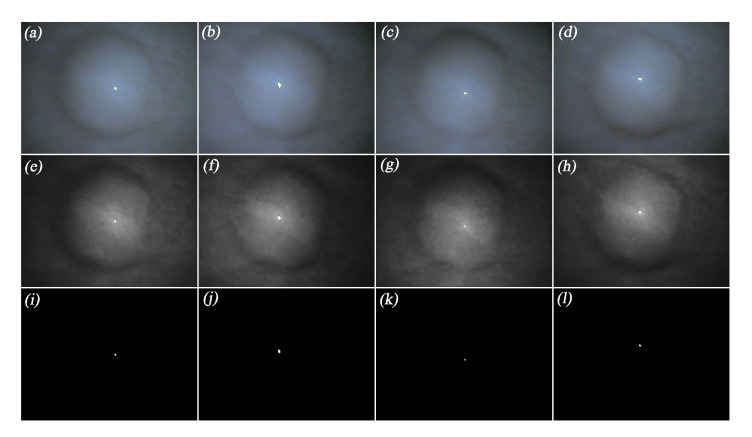
Photometric stereo images, illuminance maps, and detected specular highlight regions in the experiment. (**a**–**d**): captured photometric stereo images (after distortion correction). (**e**–**h**): illuminance maps of photometric stereo images in the experiment. (**a**–**l**): detected specular highlight regions in the experiment.

Based on the experimental configuration described in Section 6.2, we captured four photometric stereo images under the illumination of four different light sources, as shown in the first row of Figure 12. Then, the illuminance maps of the photometric stereo images were obtained by using the camera calibration parameters, as shown in the second row of Figure 12. After that, the specular highlight regions in the illuminance maps were detected by means of saturation and intensity, as shown in the third row of Figure 12.

#### 6.3.2. Depth Estimation of the Specular Highlight Points

**Figure 13:**
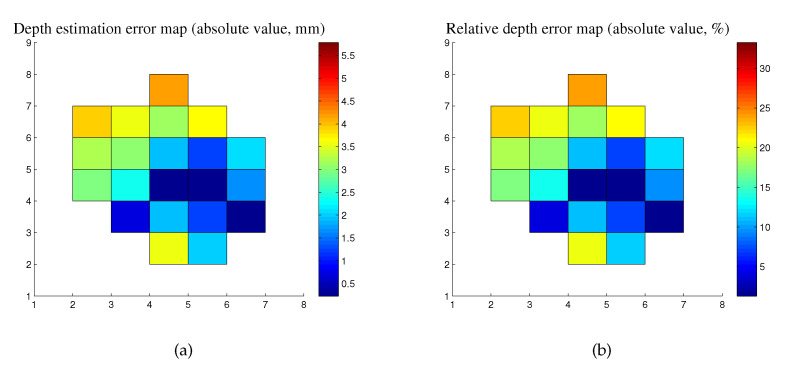
Depth estimation error distribution of the specular highlight region. (**a**) Depth estimation error map (absolute value). (**b**) Relative depth error map (absolute value).

For all pixels in the detected specular highlight regions, we implemented the proposed depth estimation method. For comparison with the simulation, here, we only discuss the results of the depth estimation of pixels in the specular highlight region of Figure 12a. The depth estimation error distribution is shown in Figure 13.

According to the obtained results, the pixel at the centroid of the specular highlight region has the minimal depth estimation error 0.2830 mm and relative depth error 1.6272%, which comes from the estimated depth −17.1100 mm and ground truth −17.3930 mm. The RMSE of the depth estimation of all pixels in the specular highlight region is 3.1892 mm, and the corresponding relative depth error is 18.3361%.

From a quantitative viewpoint, the depth estimation errors of the specular highlight region in the experiments and simulation show a similar distribution, but for pixels that are not at the center of the specular highlight region, the depth estimation errors are much more significant and not suitable for the subsequent depth map reconstruction. To meet the need of the practical application, the points with minor depth estimation need to be chosen from the specular highlight region. According to our results in the simulation and experiment, the centroid of the specular highlight region can be a good candidate.

#### 6.3.3. Depth Map Reconstruction of the Object Surface

**Figure 14:**
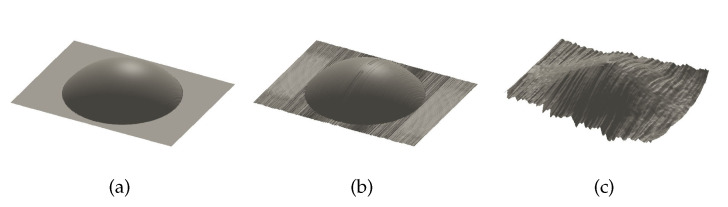
Comparison of the results in the simulation and experiment. (**a**) Ground truth depth map of the object surface. (**b**) Reconstructed depth map based on the centroid pixel of the specular highlight region in the simulation. (**c**) Reconstructed depth map based on centroid pixel of the specular highlight region in the experiment.

In the subsequent depth map reconstruction test, we used the depth estimation result of the specular highlight points in Section 6.3.2 as the boundary condition of the forward upwind scheme in Section 4.3. The centroid pixel of the specular highlight region, i.e., the specular highlight point with minimal depth estimation error, was used as the seed point of depth map reconstruction. The experiment result is compared with the ground truth depth map and the simulation results in Figure 14 and Table 3.

The reconstructed depth map of the object surface in the experiment has a similar shape as the ground truth depth map; the RMSE of the depth reconstruction of the object surface is less than 0.6 mm, and the relative RMSE of the depth reconstruction of the object surface is less than 3%. For general screenings in capsule endoscopy, this precision of the depth map reconstruction can be a good accessory means for polyp diagnosis.

## 7. Conclusions

This paper proposes a photometric stereo-based depth map reconstruction method for monocular capsule endoscopy. By combining the specular property and Lambertian property under different illumination conditions, the depth of the specular highlight point is estimated, and the depth map of the whole object surface is reconstructed by a forward upwind scheme based on photometric image ratio equations. The results of the simulations and experiments show that the proposed method can provide sufficient precision in the depth estimation of specular highlight points and the depth map reconstruction of the object surface. When the centroid of the specular highlight region is used as the seed point of the forward upwind scheme, the precision of depth map reconstruction can meet the need of the practical application.

For future works, more challenging problems will be considered both in theoretical and experimental terms. For theoretical modeling, a more precise reflection model of a non-ideal Lambertian surface will be considered to improve the depth estimation of the specular highlight. For experiments, more comparable experiments will be verified not only by the camera, but also by MRI and CT devices. For object surfaces with more practical reflection properties, more complex phantom structures [36,37] and experimental techniques [38,39] will also be considered in experiments.

## Figures and Tables

**Table 1:** RMSEs of the depth map reconstruction of the object surface in the simulation.

Depth Estimation Error of Seed Point (mm)		RMSE of Reconstructed Depth Map of Object Surface (mm)	Relative RMSE of Reconstructed Depth Map of Object Surface
Minimal error	0.0488	0.0922	0.4545%
RMSE of specular region, positive	0.9355	1.6483	8.1280%
RMSE of specular region, negative	−0.9355	1.7843	8.7982%

**Table 2:** Calibrated intrinsic parameters of the endoscopic camera.

Component	Estimation
$f_{x}$	564.9467
$f_{y}$	564.9467
$x_{0}$	322.4128
$y_{0}$	263.3339
*s*	0.0

**Table 3:** Comparison between the simulation result and the experimental result.

Type	Depth Estimation Error of Seed Point (mm)	RMSE of Reconstructed Depth Map of Object Surface (mm)	Relative RMSE of Reconstructed Depth Map of Object Surface
Simulation	0.0488	0.0922	0.4545%
Experiment	0.2830	0.5145	2.5730%
